# Predicting the potential distribution change of the endangered Francois' langur (*Trachypithecus francoisi*) across its entire range in China under climate change

**DOI:** 10.1002/ece3.11684

**Published:** 2024-07-10

**Authors:** Yaqiong Wan, Luanxin Li, Jiang Zhou, Yue Ma, Yanjing Zhang, Yan Liu, Jiaqi Li, Wei Liu

**Affiliations:** ^1^ The State Environmental Protection Key Laboratory on Biodiversity and Biosafety, Nanjing Institute of Environmental Sciences Ministry of Ecology and Environment Nanjing China; ^2^ School of Karst Science Guizhou Normal University Guiyang China

**Keywords:** anthropogenic pressures, climate change, Francois' langur, MaxEnt model, potential distribution

## Abstract

The Francois' langur (*Trachypithecus francoisi*) is a rare primate species indicated as endangered and distributed in karst areas in northern Vietnam and southwestern China. However, research limited to specific nature reserves or sites has hampered holistic conservation management. A comprehensive map of the potential distribution for the Francois' langur is essential to advance conservation efforts and ensure coordinated management across regions. Here, we used 82 occurrence records of Francois' langur surveyed in Guangxi, Guizhou, and Chongqing from 2017 to 2020, along with 12 environmental variables, to build the potential habitat model under current and future climate (2030, 2050, 2070, and 2090s) using maximum entropy models (MaxEnt). Our results indicated that (1) precipitation‐ and temperature‐associated bioclimatic variables contributed the most to the distribution of Francois' langur. Vegetation, water sources, and anthropogenic variables also affected its distribution; (2) a total of 144,207.44 km^2^ of potential suitable habitat across the entire range in China was estimated by the current model. Moderate‐ and high‐suitability habitats accounted for only 23.76% (34,265.96 km^2^) of the predicted suitable habitat and were mainly distributed in southwest Guangxi, east of Chongqing, and the border between Guizhou and Chongqing; (3) the suitable habitats of Francois' langur will contract considerably under future climate change, and the habitat centroid will move in the southeast direction with a shifting distance of approximately 2.84 km/year from current to 2100. The habitat prediction of Francois' langur and the main drivers proposed in this study could provide essential insights for the future conservation of this endangered species. The existing distribution areas should be monitored and protected, but conservation beyond existing habitats should also be a focus of effort, especially in future expansion areas. This would ensure effective and timely protection under climate change and anthropogenic pressures.

## Graphical abstract

This study shows the potential suitable habitat of Francois' langur in China under current and future climates using maximum entropy models. The habitat prediction and the main drivers proposed in this study can provide essential insights for the future conservation of this endangered species. And this will ensure effective and timely protection under climate change and anthropogenic pressures.

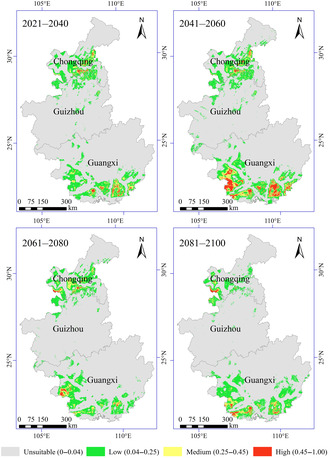

## 1 INTRODUCTION

Climate change is one of the most significant contemporary threats to biodiversity worldwide (Isaac & Williams, 2007; Jing et al., 2022). It is already affecting species and ecosystems globally, and these effects are projected to become more rapid and extensive (Pecl et al., 2017; Trew & Maclean, 2021; Wilkening et al., 2019). The impact of human activities (e.g., habitat destruction, overexploitation, and the introduction of non‐native species) is also a critical driver of species distribution changes. The negative effects of anthropogenic activities have drastically increased globally since the 1970s (Di Marco et al., 2014), likely affecting habitat connectivity and resource availability (Doherty et al., 2021). Consequently, the habitats of numerous species have shrunk or been lost (Tucker et al., 2018; Wang et al., 2019). Human disturbance interacts with ongoing climate change, which affects the distribution of some species, especially rare and endangered species like primates. For example, Fernández et al. (2022) organized into 12 threat categories of primates, including nine human disturbances, genes, diseases, and climate change and severe weather. At least three different threat types affected all 23 species groups: seven groups were affected by three to six threat types (e.g., Daubentoniidae, Cheirogaleidae, Lemuridae, Perodicticinae, Saimiriinae, Lepilemuridae, and Pitheciinae), 13 groups were affected by seven to nine threat types (e.g., Aotinae, Atelinae, Cebinae, Hylobatidae, Indriidae, Ponginae, Alouattinae, Callicebinae, Galagidae, Lorisinae, Callitrichinae, Homininae, and Tarsiidae), and three groups were affected by 10 or more threats (e.g., Cercopithecini, Papionini, and Colobinae). Many species respond to changing environments by shifting their geographical ranges for survival or going extinct (Chen et al., 2011; Parmesan, 2006).

Francois' langur (*Trachypithecus francoisi*) is an endangered species endemic to the limestone forests of the tropical and subtropical zones of northern Vietnam and southwestern China (Nadler et al., 2004). The current estimated global population is approximately 2000 individuals (Wang et al., 2011; Zeng et al., 2013). In China, the distribution of Francois' langur was largely restricted to areas characterized by karst topography at altitudes of 120–1800 m in Guangxi, Guizhou, and Chongqing (Han et al., 2021). Karst landforms were formed by dissolving porous soluble bedrock, namely limestone. These areas exhibit unique characteristics that set them apart from mountain forests, including steep cliffs covering approximately 10%–20% of the area. Karsts are generally harsh and dry, with rainwater draining quickly (Blair et al., 2021), an absence of surface water (Huang, 2002), and relatively less fertile yet more diverse vegetation (Long & Trien, 2019; Zhou et al., 2013). Due to forest loss, habitat destruction, and fragmentation, some Francois' langur populations have been locally extirpated from some parts of their historic range (Li et al., 2007; Niu et al., 2016; Wang et al., 2011; Wen et al., 2010; Zeng et al., 2013). For instance, this species has become restricted to only five isolated sites in Guizhou (Hu et al., 2011). Francois' langur is listed as an endangered species on the International Union for Conservation of Nature Red List (IUCN, 2023) and a first‐grade protected wildlife species in China. Despite this scenario, little was known about its habitat use and influencing factors. Researchers have studied this species' roost selection and behavioral ecology. However, these studies were limited to specific nature reserves or locations (Hu et al., 2011; Li et al., 2020; Liu & Bhumpakphan, 2020; Zhou et al., 2013, 2018).

A comprehensive potential habitat map for the Francois' langur across its entire range in China remains a gap in our understanding of this species. It hampers effective, integrated, and holistic conservation. Species distribution models (SDMs) are appropriate solutions to such challenges that can overcome sampling problems and generate reliable, consistent, and transparently derived estimates over large areas (Drew et al., 2010). SDMs are suitable for a species with a narrow ecological range (Hernandez et al., 2006), such as Francois' langur. SDMs are important tools for ecology and biogeography research and are typical methods for combining specific species with niche factors (Zhang et al., 2018, 2019). Currently, SDMs mainly include maximum entropy models (MaxEnt), random forest, boosted regress trees, generalized additive models, and multivariate adaptive regression splines. Among these models, MaxEnt is a high‐performing, highly popular model. It can use widely available presence‐only data and deal powerfully with limited occurrence data and small sample sizes (Fourcade et al., 2014; Merow et al., 2013; Phillips et al., 2006; Phillips & Dudik, 2008). It combines the current distribution data of species with the background environmental variables and associates known occurrences with a set of unique environmental conditions. Then, it predicts the probability of presence for unknown locations using multiple algorithms, and evaluates the importance of environmental variables using jackknife (Yin et al., 2022). Thus, MaxEnt can be applied to well predict the distribution of Francois' langur.

In this study, MaxEnt was applied to construct the potential distributions of Francois' langur. The primary objectives were to (1) model the current potential distribution of the Francois' langur habitat in China, (2) explore the key environmental factors affecting its distribution, (3) predict its future distribution for the years 2030, 2050, 2070, and 2090, (4) and evaluate how future climate change will affect the potential distribution of this species. This study will provide suggestions and new insights for developing effective management and protection of the Francois' langur.

## 2 MATERIALS AND METHODS

### 2.1 Occurrence data of Francois' langur

**FIGURE 1:**
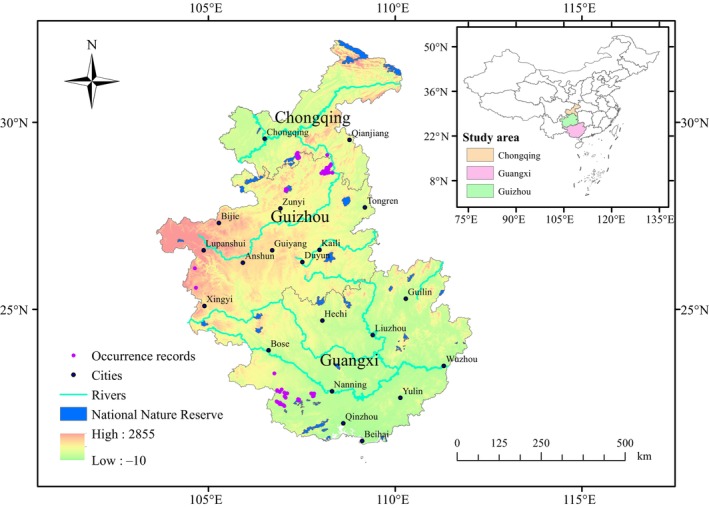
Study area and occurrence records of Francois' langur in the present study.

We conducted field surveys in all known distribution areas of the species in Guangxi, Guizhou, and Chongqing provinces, located in southwest China, with a special ecological environment of the karst ecosystem from 2017 to 2020 (Figure 1). We recorded the occurrence data based on 36 trail lines of 3 km along the paths of animals, and camera trap surveys in the centroid of 600 1 × 1 km grids, based on the method from the China Biodiversity Observation Network (Wan et al., 2020). We obtained 171 occurrence records of Francois' langur. We used the SDM Toolbox v2.4 (spatially rarefy occurrence data) of ArcGIS 10.2 to ensure that there was only one occurrence record for the species in each 1 × 1 km resolution grid cell and avoid overfitting in the models and to prevent spatial auto‐correlation of raster data (Li et al., 2022; Tu et al., 2021). After selection, 82 occurrence records of Francois' langur were identified for modeling analysis.

### 2.2 Environmental variables and data processing

We provisionally selected 26 environmental variables to build the initial models based on previous research on the habitat of Francois' langur. These variables included climate (19 bioclimatic variables), topography (elevation, slope, and aspect), vegetation (normalized Difference Vegetation Index [NDVI]), water sources (distance to rivers), and human activities (distance to road and distance to housing).

Nineteen grid‐based bioclimatic variable data were downloaded from the WorldClim (www.worldclim.com) dataset. The current climate data were obtained with a spatial resolution of 30 arc‐second (approximately 1 km^2^) (Thapa et al., 2018). Future climate data were obtained from the Beijing Climate Center Climate System Model (BCC‐CSM2‐MR) under the Coupled Model Intercomparison Project Phase 6 (CMIP6) global climate model (Shi et al., 2020; Yin et al., 2022) in four periods: 2021–2040, 2041–2060, 2061–2080, and 2081–2100. The shared socioeconomic pathways (SSPs 126, 245, 370, and 585) showed the annual CO_2_ emissions expected for 2100. We used the SSP245 pathway for the moderate scenario assumption, which generally limits warming to around 3°C by 2100. These climatic variables represented annual trends (mean annual temperature and precipitation), seasonality (annual range of temperature and precipitation), and limiting environmental factors (temperature and precipitation of a certain quarter) (Hijmans et al., 2005; Thapa et al., 2018). The 19 bioclimatic variables were all extended from temperature and precipitation based on different calculations. The 19 predictor climatic variables used in this study were tested using Pearson correlation analysis with the “Cor” function in R 4.3.0 to avoid the effects of model overfitting caused by the multicollinearity of these variables (R Core Team, 2014; Zhao et al., 2020) (Appendix S1). Variables with high correlation coefficients (|*r*| ≥ .8) and relatively low contribution rates in the initial models were removed to improve model predictability (Ji et al., 2020; Liu et al., 2022).

Three topography variables, including elevation, slope, and aspect, were derived from Digital Elevation Model data obtained from the geospatial data cloud (http://www.gscloud.cn) with a resolution of 30 m using ArcGIS 10.2. NDVI was downloaded from the Resources Environment Data Center, Chinese Academy of Sciences (http://www.resdc.cn) with a resolution of 500 m. River, road, and housing data were obtained from the National Basic Geographic Information System, and the Euclidian distance tool of ArcGIS 10.2 was used to calculate the distance between each grid cell layer and the nearest river, road, and housing.

Consequently, five predictor climate variables were retained, namely, the Mean Diurnal Range (Mean of monthly [max temp–min temp]) (Bio2), Min Temperature of Coldest Month (Bio6), Temperature Annual Range (Bio7), Precipitation Seasonality (Coefficient of Variation) (Bio15), and Precipitation of Coldest Quarter (Bio19). Additionally, elevation, slope, aspect, NDVI, distance to river, distance to road, and distance to housing were also used in the model prediction (Table 1). We assumed that all variables in the future climate models, except for the climate factors, would be roughly unchanged. All variables selected for modeling were projected to WGS_1984_UTM_Zone_48N and resampled to 30 arc‐second (approximately 1 km^2^) to facilitate spatial analysis within China using the spatial analysis tools in ArcGIS 10.2.

**TABLE 1:** Environmental variables included in the analysis and their percentage contribution and permutation importance in predicting the current distribution of Francois' langur.

Variables	Description	Percent contribution (%)	Permutation importance (%)
Bio2	Mean of monthly (max temp–min temp)	15.6	9.5
Bio6	Min temperature of coldest month	20.4	7.1
Bio7	Temperature annual range	7.4	15.3
Bio15	Precipitation seasonality (coefficient of variation)	5.1	10.7
Bio19	Precipitation of coldest quarter	14.9	34
Ele	Elevation	4.4	2.3
Slop	Slope	2.3	4.1
Asp	Aspect	0.7	0.3
NDVI	Normalized Difference Vegetation Index	3.2	1.2
Dis_river	Distance to nearest river	2	2.5
Dis_road	Distance to nearest road	20.6	11.7
Dis_house	Distance to nearest housing	3.4	1.4

### 2.3 MaxEnt modeling

We used the open‐source software package MaxEnt v.3.4.4 (http://biodiversity informatics.amnh.org/open_source/maxent/) to produce the distribution ranges for Francois' langur in the different climate scenarios (Phillips et al., 2006). Francois' langur occurrence records and environmental variables were imported into MaxEnt. All models were set as random seeds and used 75% of the record data as the training set and the remaining 25% as the test set with 10 replicates and bootstrap as the replicated run type (Wang et al., 2020). The number of modeling iterations was set at 10,000 to give the models adequate time for convergence (Phillips et al., 2006). The jackknife test procedure was used to evaluate the relative importance of each environmental variable. The response curves of the environmental variables were used to test the correlation between the variables and the occurrence probability of Francois' langur. Logistic was selected to output results. The default options were used for the other parameters.

### 2.4 Model evaluation and validation

Receiver operating characteristic (ROC) curve analysis is an effective method for evaluating the accuracy of SDMs (Liu et al., 2022; Wang et al., 2007). We used the area under the ROC curve (AUC), a threshold‐independent discrimination metric that represents the probability that a random presence or absence is correctly assigned by the model (Phillips et al., 2006). The AUC values range from 0.5 to 1.0, with an AUC > 0.9 indicating excellent performance (Janitza et al., 2013). Furthermore, the null model approach was used for modeling validation (Raes & ter Steege, 2007). We developed frequency histograms of the expected AUC values by randomly drawing points without replacement from the geographical area of Francois' langur. We modeled these with Maxent under the same environmental conditions as the species. We compared the AUC values of each model to a one‐sided 95% confidence interval derived from the null distribution of the average AUC value (Deka et al., 2023).

The mean value of the 10 replicates from the model was taken as the distribution result, and the value represents the habitat suitability index, which was between 0 and 1. The ASCII output file from MaxEnt was transformed into a raster layer and reclassified using the spatial analyst tool. Currently, the classification of suitable habitat areas is largely based on experience, and there is no unified standard (Sun et al., 2020). We used Jenks' natural breaks classification in ArcGIS combined with maximum training sensitivity plus specificity (Max TSS), balance training omission, predicted area, and threshold value (average TPT) in the MaxEnt output to reclassify the data into four suitability areas for Francois' langur: high habitat suitability, moderate habitat suitability, low habitat suitability, and unsuitable habitat. The spatial analysis tool in ArcGIS 10.2 was used to calculate the areas and proportions of each suitability area.

### 2.5 Changes in potential distribution and centroid shifts

Binary suitable or unsuitable maps were produced using ArcGIS 10.2 to analyze the changes in centroid and potential distributions based on the threshold of the unsuitable area and construct the core distribution shifts and potential area changes under each future climate model. Four distribution changes were determined (e.g., contraction, expansion, no change, and no occupancy) by comparing the suitable/unsuitable potential distributions in different time periods (Jose & Po, 2020; Liu et al., 2022). Moreover, because the potential distribution of Francois' langur was scattered and its outline was irregular, centroid shift analysis was conducted based on the potential distribution under different periods using SDMtoolbox v2.4 (Brown, 2014) to visually show the changing trends of the potential core distribution of the species.

## 3 RESULTS

### 3.1 Model performance

Predicting suitable habitats for Francois' langur with MaxEnt resulted in high model performance. The AUC metric for the 10 replicate runs was 0.973 ± 0.006 (mean ± SD) (Appendix S2), indicating a high level of predictive performance in the current period. For future climate models of the Francois' langur distribution, the obtained AUC values in 2021–2040, 2041–2060, 2061–2080, and 2081–2100 were 0.977, 0.977, 0.974, and 0.975, respectively. All AUC values exceeded the 0.9 AUC threshold, indicating good model performance. Additionally, the null model confirmed that the model outperformed a random model significantly, with an average AUC value of 0.783.

### 3.2 Environmental variable contributions

**FIGURE 2:**
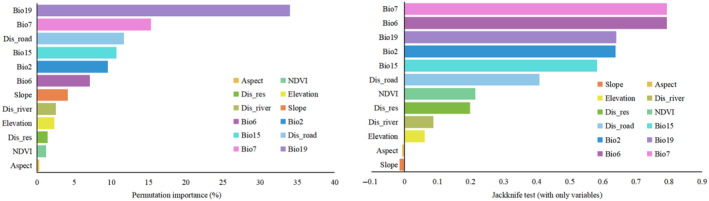
Relative predictive power of different environmental variables based on the jackknife test and permutation importance in maximum entropy models for Francois' langur.

The jackknife analysis showed the contribution of each variable and their permutation importance in predicting the habitat of Francois' langur. The current prediction was mainly affected by Dis_road (20.6%), Bio6 (20.4%), Bio2 (15.6%), Bio19 (14.9%), Bio7 (7.4%), and Bio15 (5.1%) in decreasing the percentage of their contribution toward the prediction model. All other variables contributed less than 16% (Table 1). When considering the permutation importance, Bio19 (34%), Bio7 (15.3%), Dis_road (11.7%), Bio15 (10.7%), Bio2 (9.5%), and Bio6 (7.1%) showed the highest percentage. The cumulative contribution of these variables was more than 88%, the main factors contributing to the MaxEnt model (Figure 2).

### 3.3 Response to environmental variables

Species response curves revealed the relationship between environmental variables and the probability of species presence, indicating biological tolerance for target species and habitat preferences. Based on the acquired species response curves (Appendix S3), Francois' langur prefers habitats with a mean diurnal range (mean of monthly [max temp–min temp]) (Bio2) lower than 6°C, min temperature of the coldest month (Bio6) of 9–11°C, temperature annual range (Bio7) of 21–23°C, precipitation seasonality (coefficient of variation) (Bio15) lower than 65 mm, precipitation of coldest quarter (Bio19) from 70 to 80 mm. Meanwhile, the probability of Francois' langur presence increases with increasing distance to the road in the range of 0–15 km, distance to housing in the range of 0–30 km and NDVI from 0 to 0.8. The probability of Francois' langur presence decreases as the distance to the river increases.

### 3.4 Current potential distribution

**FIGURE 3:**
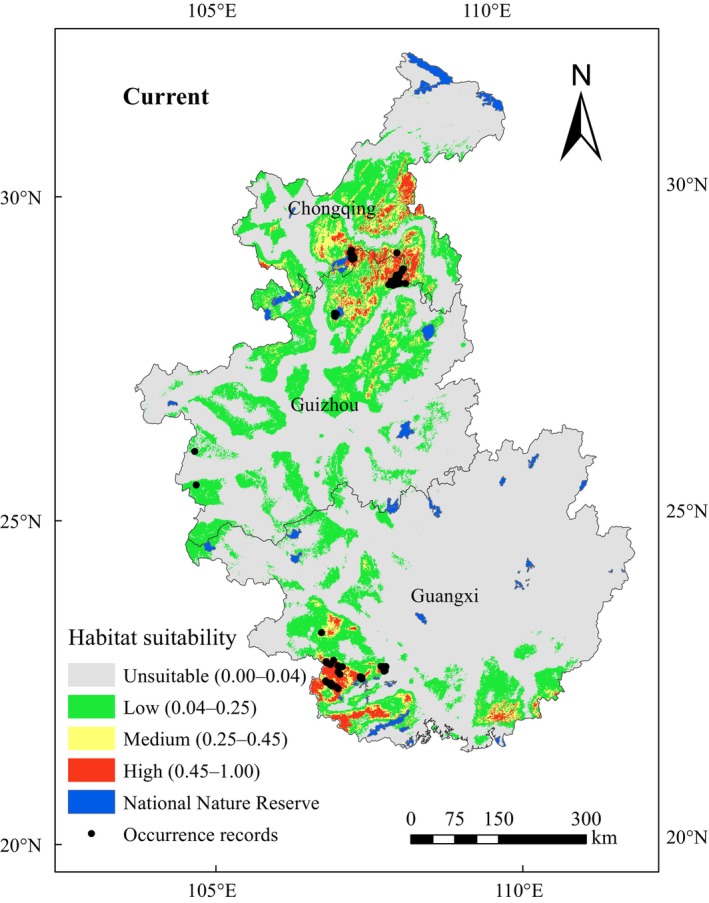
Current potential distribution map of Francois' langur.

A map of the current habitat suitability for Francois' langur based on MaxEnt model predictions is shown in Figure 3. This map was based on the 12 environmental variables and the current occurrence data of the species. All 82 occurrence records fell within the predicted suitable area, again indicating that the model performed well and produced an adequate evaluation. The total suitable habitat area was predicted to be 144,207.44 km^2^ (29.09% of the study area), of which the highly suitable area (0.45–1) covered 13,108.55 km^2^ (9.09% of the total suitable area). The moderately suitable area (0.25–0.45) covered 21,157.41 km^2^ (14.67% of the total suitable area), and the poorly suitable area (0.04–0.25) covered 109,941.49 km^2^ (76.24% of the total suitable area). The highly suitable areas were located in a small part of southwest Guangxi, east of Chongqing, and on the border between Guizhou and Chongqing. The moderately suitable habitats were close to the highly suitable ones, with different expansions. Less suitable areas, which were fragmented, were mainly located in Guizhou and some parts of Chongqing, except in the northern area and southwest of Guangxi (Figure 3).

### 3.5 Future changes in suitable habitats

**FIGURE 4:**
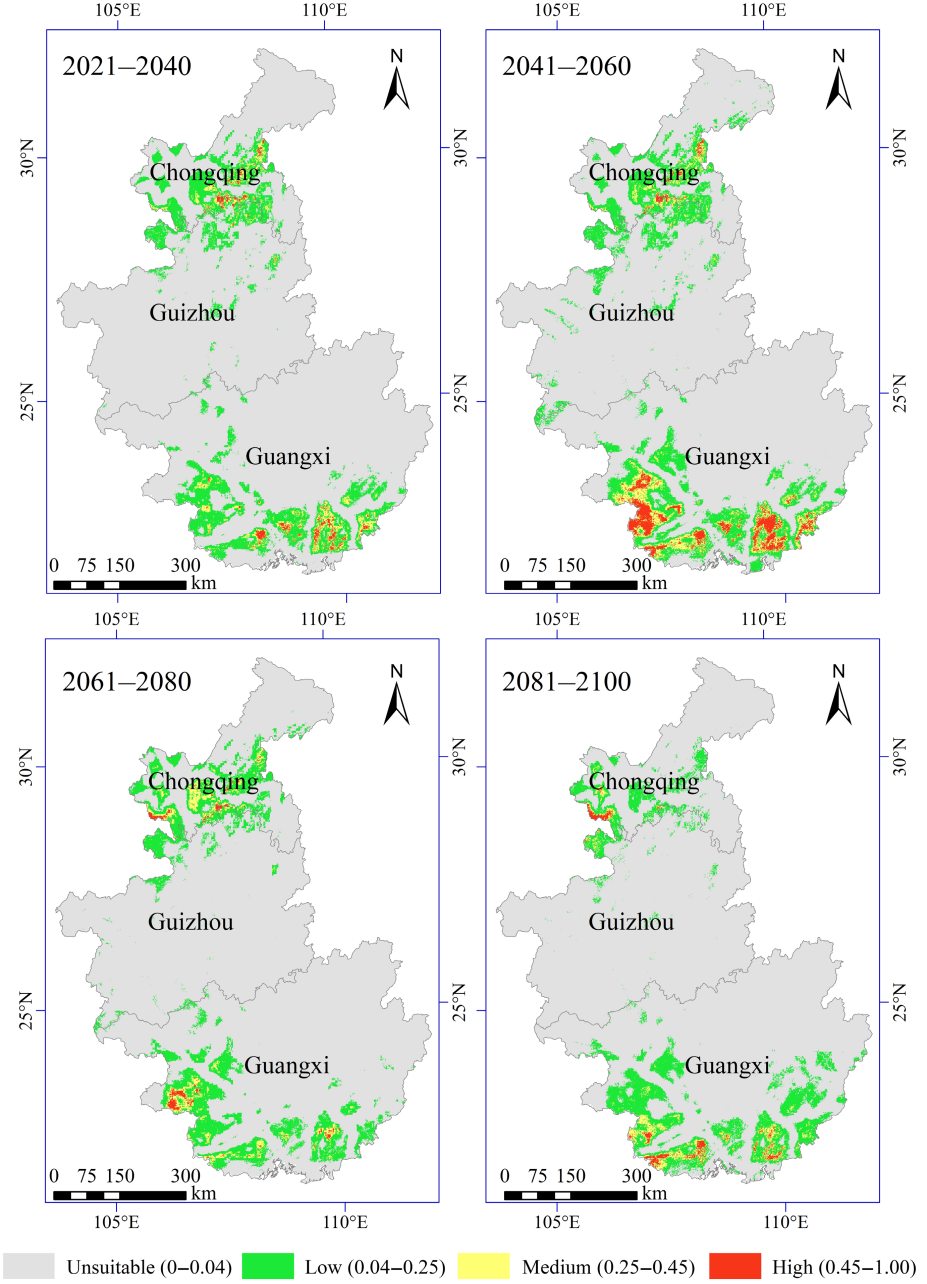
Predicted future distributions of periods 2021–2040, 2041–2060, 2061–2080, and 2081–2100 for Francois' langur.

**FIGURE 5:**
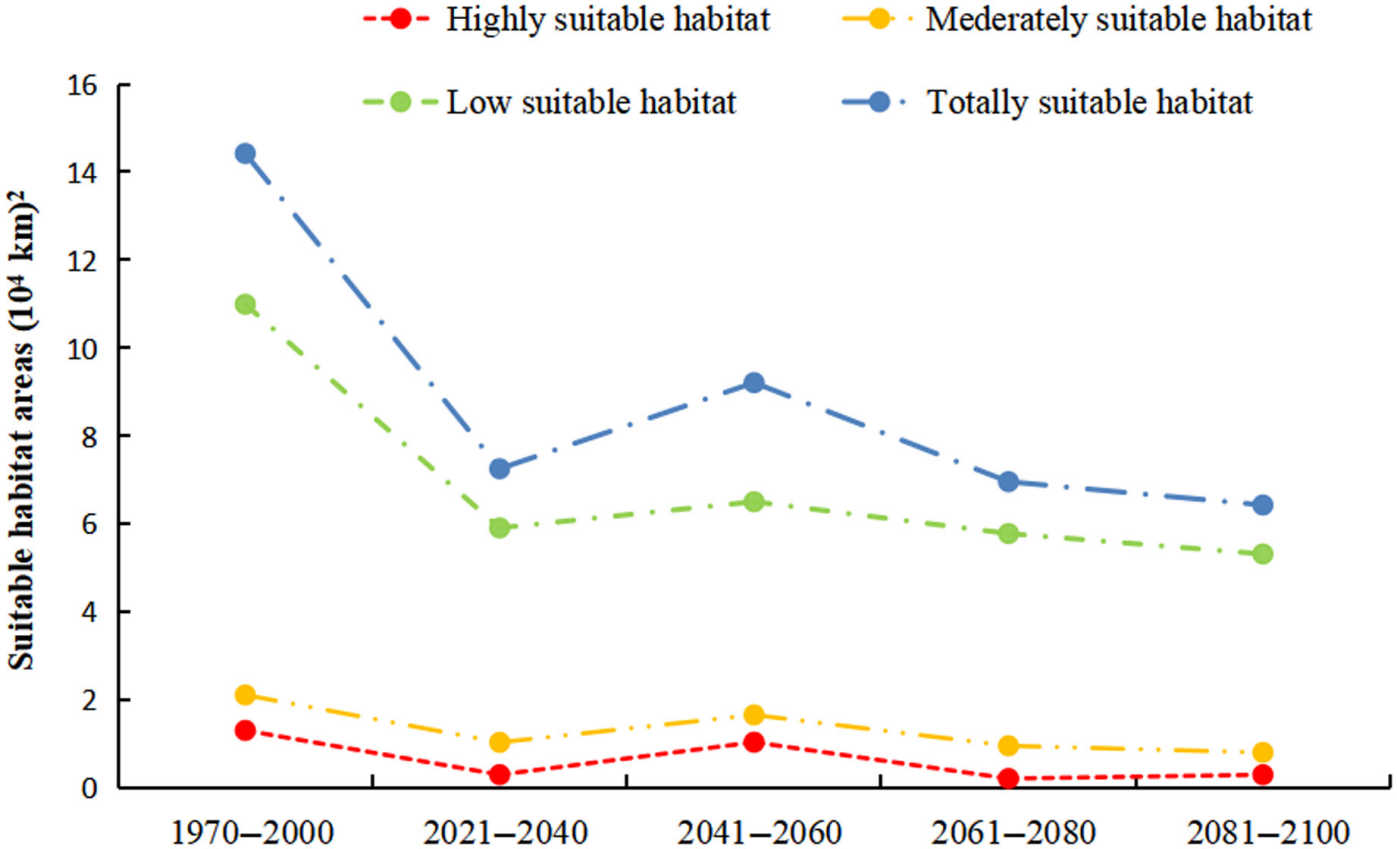
Suitable habitat areas of Francois' langur in all different periods.

The area of the potential geographical distribution of Francois' langur was predicted to decrease compared with the current situation under future climate conditions (2021–2040, 2041–2060, 2061–2080, and 2081–2100) in the SSP245 climate scenario. Habitat suitability maps for future climate models are shown in Figure 4. The high potential suitable area for Francois' langur would decrease by 76.73% by 2021–2040. Similarly, the moderately suitable area would decrease by 50.87%, and the low‐suitable area would decrease by 46.22%. However, the highly, moderately, and poorly suitable areas would only decrease by 20.47%, 21.42%, and 40.81%, respectively, by 2041–2060. This result revealed an increase in the suitable area for Francois' langur compared with 2021–2040. The highly suitable area would decrease by 83.46% by 2061–2080, the medium suitable area would decrease by 54.56%, and the low‐suitable area would decrease by 47.39%. A similar pattern was also observed for 2081–2100, where the highly, moderately, and poorly suitable areas decreased by 76.85%, 61.73%, and 51.62%, respectively (Figure 5).

### 3.6 The centroid distribution shifts

**FIGURE 6:**
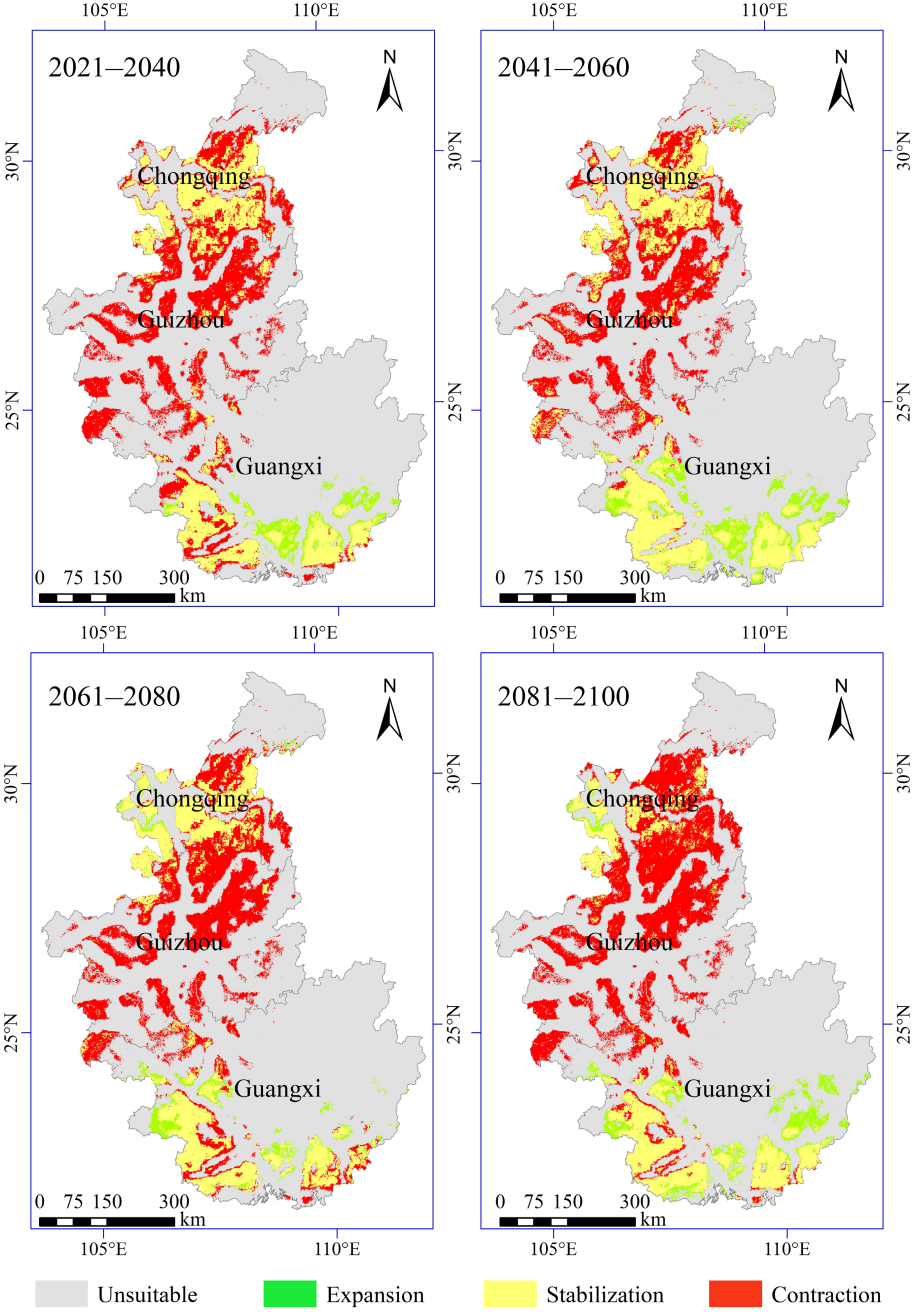
Potential distribution changes in the future (2021–2040, 2041–2060, 2061–2080, and 2081–2100) compared with current for Francois' langur.

**FIGURE 7:**
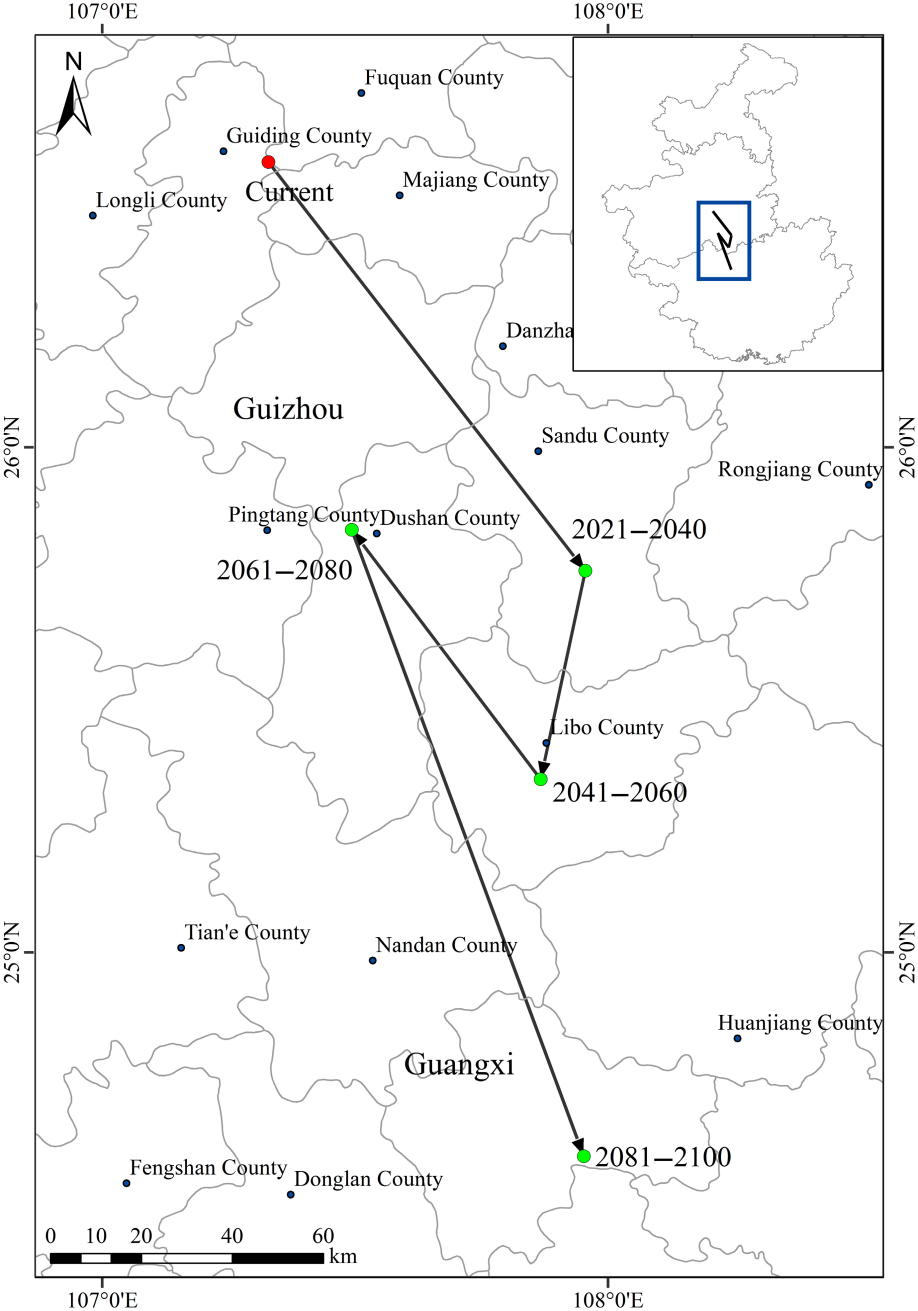
Centroid change in the potential distribution of Francois' langur from 2021 to 2100. The distance of the centroid shifts was 109.43, 145.99, 82.26, and 227.09 km in 2021–2040, 2041–2060, 2061–2080, and 2081–2100, respectively, compared with the current.

The change in the potential distribution of Francois' langur from 2021 to 2100 is shown in Figure 6. These models predicted that future global change would promote the contraction of the potentially suitable habitats for Francois' langur, decreasing the total suitable habitats. Particularly, the most suitable habitats in Chongqing and Guizhou would contract sharply. Francois' langur would expand slightly in southwestern Guangxi and western Chongqing. The potential core distribution of Francois' langur would move southeast from southern Guizhou to northern Guangxi under future climate conditions. Furthermore, the distances of the centroid shifts were 109.43, 145.99, 82.26, and 227.09 km in 2021–2040, 2041–2060, 2061–2080, and 2081–2100 scenarios, respectively, compared with the current (Figure 7).

## 4 DISCUSSION

### 4.1 Influence of environmental variables

In Francois' langur ecology, habitat selection and use have been important research topics for researchers. The factors of habitat selection have been divided into biological, geographical, and anthropogenic factors (Han et al., 2023). Our results suggested that bioclimatic variables made the largest contribution to the distribution of Francois' langur, vegetation, water source, and anthropogenic variables were also important factors influencing its distribution. As demonstrated by numerous previous studies, the distribution of Francois' langur was largely restricted to areas characterized by karst topography, limestone cliffs, and caves with mixed conifer‐broad‐leaf forests, and these areas provided quality food resources and cover for predator avoidance (Wang et al., 2011; Wen et al., 2010; Zeng et al., 2013). Various factors, including temperature, precipitation, and vegetation, concurrently influenced its distribution (Enstam & Isbell, 2004; Han et al., 2011).

According to our model prediction, precipitation‐associated bioclimatic variables were the most important habitat predictor for Francois' langur, followed by temperature‐associated bioclimatic variables. Numerous studies have shown that in recent years, the frequency of precipitation in southwest China has decreased while the intensity, total precipitation, and occurrence of extreme rainstorms have increased (Ding, 2014; Feng et al., 2012; Lu et al., 2022). This has directly threatened the habitat of the Francois' langur by raising the water level of the rivers and flooding the original valley area with food and shelter caves. In addition, temperature and precipitation have a great influence on the growth rates of plant species. Fruits, seeds, and especially young leaves are the main food of Francois' langur. Therefore, temperature and precipitation indirectly determine its distribution by affecting the availability of the potential food resources (Huang et al., 2008; Zhou et al., 2006, 2011). The temperature and precipitation characteristics for the most suitable distribution are consistent with the climatic condition of the extant range of Francois' langur based on the response curves of bioclimatic variables in our study (Han & Hu, 2010; Li et al., 2019; Niu et al., 2016).

Vegetation‐related variable, the NDVI, was another important variable. Francois' langur prefers areas with relatively high NDVI, consistent with Han et al. (2023), Niu et al. (2016), Zeng (2011), and Zeng et al. (2013). These researchers showed that this species' habitat was mainly in the valley areas on both sides of the rivers, covered by evergreen broad‐leaf forests with higher tree canopy density and vegetation cover, which provided the availability of better food, water resources, and sleeping sites (Han et al., 2011; Hu, 2011; Wang et al., 2011). Meanwhile, distance to rivers and altitude were the less important variables, as the elevation of Francois' langur distribution ranges from 300 to 1800 m, with seasonal changes in climate and food (Li et al., 2015, 2019).

For the anthropogenic variables, distance to roads (including railways) was more important than distance to housing. Most previous studies have assessed the impact of linear infrastructure on wild animals and have typically shown that distribution decreases as road density increases (Clauzel et al., 2013, 2015). Roads are likely to act as barriers to wildlife movement (Coffin, 2007; Zhu et al., 2011). Therefore, roads may harm the activity and distribution of Francois' langur. In this study, the distance to roads was positively correlated with the occurrence probability of Francois' langur. However, the distance to housing had a limited influence on the distribution prediction of this species. This finding aligns with some published researches (Han et al., 2023; Wang et al., 2011; Zeng et al., 2013), which reported that Francois' langur has become accustomed to human disturbances, especially crops grown on agricultural land that may supply food resources.

### 4.2 Characteristics of the current potential distribution

Our model estimated a potentially suitable habitat of 144,207.44 km^2^ for Francois' langur across the range. However, Hu et al. (2011) showed that this species was restricted to only five isolated sites (Dashahe, Baiqing, Mayanghe, Kuankuoshui, and Yezhong) in Guizhou. There was a total area of approximately 912 km^2^, and Chen (2006) estimated the potential habitat in Guangxi at approximately 3216.23 km^2^. Han and Hu (2010) confirmed the distribution area in Chongqing Jinfo Mountain was approximately 50 km^2^. Thus, our model predicted that the distribution area in three provinces (Guizhou, Guangxi, and Chongqing) was much larger than that predicted in the above studies. These previous studies were based on survey records or estimates on a small scale, such as one or more nature reserves. Importantly, our model analysis was based on a machine learning algorithm, species records from the entire range in China, and environmental variables, including climatic factors. Furthermore, our model prediction for the Francois' langur was based on a larger and more comprehensive scale than prior methods (Chen, 2006; Han et al., 2011, 2023; Hu et al., 2011).

Numerous studies have recognized that the MaxEnt algorithm produces highly accurate predictions over various species and geographic regions (Elith et al., 2006; Hernandez et al., 2006). The current potential distribution in our study was larger than the historical distribution areas of Francois' langur (Jiang et al., 2015; Smith et al., 2010). Our results can provide a scientific reference for further field surveys on the distribution of Francois' langur in China. In addition, our distribution map indicated that large potential distribution areas were found outside the national protected areas (Figure 3). Thus, this species conservation needs a greater focus on creating new protected areas or other effective area‐based conservation measures (OECMs). OECM is an emerging and critical international issue that has become an indispensable supplement to protected areas and has important practical significance for promoting in‐situ conservation and diversifying conservation methods (Wang et al., 2021). Especially for Francois' langur populations, such as Changliuxiang populations, living alongside the local communities, OECMs should be considered a good protection strategy. The sustainable use of natural resources, alternative livelihoods, and co‐developing strategies should be supported to improve human‐primate coexistence in shared landscapes (Fernández et al., 2022).

From the range of suitable areas, our model predicted 34,265.96 km^2^ of moderate‐ and high‐suitability habitats for Francois' langur. These habitats, primarily located in southwest Guangxi, east of Chongqing, and the Guizhou‐Chongqing border, were nearly 10 times the area studied in previous surveys (Chen, 2006; Han & Hu, 2010; Hu et al., 2011). The moderate‐ and high‐suitability habitats only account for 23.76% of predicted suitable habitats but should be of great concern. We predicted that these habitats covered almost all occurrence records of this species. These areas should be protected as critical areas for Francois' langur. Some moderate‐ and high‐suitability habitats are covered by current protected areas, such as Chongqing Jinfo Mountain, Guizhou Mayanghe, Kuankuoshui, Guangxi Nonggang, and Chongzuo National Nature Reserves. Still, human activities, such as grazing, deforestation, and even illegal hunting in these protected areas, threaten this primate. Therefore, the current protected areas must be strictly managed to safeguard preferred habitats. However, most moderate‐ and high‐suitability habitats are outside protected areas. We need to pay attention to these areas and take measures. For example, the Dashahe in Daozhen County on the northern border of Guizhou Province has a Francois' langur population, and this area borders the Jinfo Mountain Reserve of Chongqing. A habitat corridor between Dashahe and Jinfo can be built or merged into a national nature reserve.

Moreover, the low‐suitable habitat was distributed over a larger area but as fragmentation. This result is consistent with Guan et al. (2022) and Niu et al. (2016), who suggested that Francois' langur habitats presented severe fragmentation, complex patch shape, weak patch aggregation, and decentralization. Meanwhile, they typically showed that habitat areas decreased and further fragmented with increasing human activities. For this fragmented, low‐suitable area predicted by our model, we should put considerable effort into surveying population and habitat status, then develop strategies to reduce human activities and connect the fragmented habitats as much as possible.

### 4.3 Distribution change in the future

Our results indicate that, compared with the current potential habitat, the suitable habitats of Francois' langur will decrease in all future periods under climate change. The temperature in southwest China is expected to continue increasing in the future climate prediction, and show a more obvious difference in the increase between the northern and southern regions (Yang et al., 2022; Zhang, 2020). Climate change will lead to changes in species habitats, such as increased precipitation and more extreme precipitation events (Feng et al., 2023; Yue, 2022). The suitable habitat area will contract considerably, especially in the medium‐ and high‐suitability areas along the border between Guizhou and Chongqing, from 2021 to 2100. Moreover, most patchy areas of low suitability will cease to exist. Previous studies have indicated habitat loss and fragmentation from encroachment for settlements and farming were the principal threats to primates (Chetry et al., 2021). Climate change and land use/cover change could reduce suitable habitats for primates and increase their extinction risk (Carvalho et al., 2019; Deb, 2016). Fernández et al. (2022) also agreed that agriculture, human disturbance, and climate change were more likely to be considered at risk of extinction for primates. In addition, our results are consistent with the description that primates, like other large mammals, depend highly on a complete forest ecosystem for their food and security requirements. Therefore, forest loss and habitat fragmentation will accelerate local population extinctions (Guan et al., 2022). For Francois' langur, climate change and habitat fragmentation caused by anthropogenic disturbance are the most important factors in changing the distribution of this species.

From the perspective of future distribution, the future distribution area of Francois' langur can be divided into two geographical populations: north and south. The different geographical populations of Francois' langur significantly differ in habitat and food composition. For example, the Guangxi population mainly lives in the northern tropical seasonal rainforest at 300–600 m altitude. The landform is primarily a peak cluster depression and valley. At the same time, the Guizhou and Chongqing populations mainly live in 600–1800 m subtropical evergreen broad‐leaved forest and mixed coniferous forest, and peaks, valleys, and troughs primarily characterize the terrain (Han et al., 2013). The proportion of fruit intake in the food composition of the Guizhou population was significantly higher than that of the Guangxi and Chongqing populations. The Guangxi population ate young leaves, while the Chongqing population ate more mature leaves (Han & Hu, 2010; Li et al., 2009; Zhou et al., 2006). These differences are related to vegetation composition and phenology in habitats of different geographical populations (Zhou & Huang, 2021). In the future, under the pressure of human disturbance and climate change, populations in different regions will tend to be patchy, and geographical isolation will prevent groups from exchanging genes, resulting in a decline in genetic diversity and even species extinction. Therefore, ecological corridors can be established for geographically close populations to expand habitat range and increase population communication, such as the Chongqing Guizhou border populations, Guangxi Daxin, and Debao populations.

### 4.4 Conservation recommendations

Our results showed that the currently predicted habitats of Francois' langur were over more than the historical areas. Still, the distribution habitats would be threatened to contract and fragment considerably in the future. We suggest that for moderate‐ and high‐suitability habitats, we should focus on (1) strengthening the management of current nature reserves to stop human destructive activities on forests, considering the creation of new nature reserves or OECMs; (2) exploring the implementation of community‐based conservation and awareness; and (3) following population status and demographic trends through regular monitoring to develop further protection strategies. For less suitable areas, we should expand the survey range to know the possible habitat and enhance habitat connectivity by establishing ecological corridors, such as the border of Chongqing and Guizhou. We must be vigilant and take timely action to stop habitat loss, considering future habitat contraction distribution.

## AUTHOR CONTRIBUTIONS

**Yaqiong Wan:** Conceptualization (lead); data curation (lead); formal analysis (equal); investigation (equal); methodology (lead); writing – original draft (lead); writing – review and editing (equal). **Luanxin Li:** Investigation (equal); writing – review and editing (equal). **Jiang Zhou:** Investigation (equal); writing – review and editing (equal). **Yue Ma:** Writing – review and editing (equal). **Yanjing Zhang:** Software (equal). **Yan Liu:** Supervision (equal); writing – review and editing (equal). **Jiaqi Li:** Supervision (equal); writing – review and editing (equal). **Wei Liu:** Supervision (equal); writing – review and editing (equal).

## CONFLICT OF INTEREST STATEMENT

There are no conflicts of interests, financial or otherwise, for any of the author.

## Supporting information


Appendix S1.

## Data Availability

Data supporting this study are provided as Supporting Information accompanying this manuscript.
