# Supplementary material for: Predicting the potential distribution change of the endangered Francois' langur (Trachypithecus francoisi) across its entire range in China under climate change
Source: Ecol Evol. 2024 Jul 10;14(7):e11684. doi: 10.1002/ece3.11684 (PMC11236436; doi:10.1002/ece3.11684)
Supplement: Supplementary file 1 — Appendix S1. [file ECE3-14-e11684-s001.zip › Supporting Information.docx]

1. 171 occurrence records of Francois' langur

| species | longitude | latitude |
| --- | --- | --- |
| Trachypithecus francoisi | 106.8540083 | 22.81737222 |
| Trachypithecus francoisi | 106.8489528 | 22.81720278 |
| Trachypithecus francoisi | 106.8540083 | 22.81737222 |
| Trachypithecus francoisi | 106.8405917 | 22.820875 |
| Trachypithecus francoisi | 106.9103333 | 22.79312778 |
| Trachypithecus francoisi | 106.9126333 | 22.79208889 |
| Trachypithecus francoisi | 106.8403167 | 22.83664444 |
| Trachypithecus francoisi | 106.8565861 | 22.82094167 |
| Trachypithecus francoisi | 106.7656833 | 23.28708889 |
| Trachypithecus francoisi | 106.7656833 | 23.28708889 |
| Trachypithecus francoisi | 106.7654722 | 23.28556111 |
| Trachypithecus francoisi | 106.9608472 | 22.8595 |
| Trachypithecus francoisi | 107.1084111 | 22.75594167 |
| Trachypithecus francoisi | 107.1061639 | 22.75717222 |
| Trachypithecus francoisi | 107.1019361 | 22.75945 |
| Trachypithecus francoisi | 107.0339861 | 22.77448611 |
| Trachypithecus francoisi | 107.0365722 | 22.77336944 |
| Trachypithecus francoisi | 107.0387083 | 22.77494167 |
| Trachypithecus francoisi | 107.0394056 | 22.77441111 |
| Trachypithecus francoisi | 107.0763528 | 22.75128333 |
| Trachypithecus francoisi | 107.0667361 | 22.63957222 |
| Trachypithecus francoisi | 107.0387889 | 22.67466111 |
| Trachypithecus francoisi | 107.0393417 | 22.67693333 |
| Trachypithecus francoisi | 107.4020028 | 22.58931111 |
| Trachypithecus francoisi | 107.4012528 | 22.58989722 |
| Trachypithecus francoisi | 107.39895 | 22.59675556 |
| Trachypithecus francoisi | 107.3969139 | 22.59682222 |
| Trachypithecus francoisi | 107.4026111 | 22.59667778 |
| Trachypithecus francoisi | 107.4017778 | 22.59966944 |
| Trachypithecus francoisi | 107.4076889 | 22.5939 |
| Trachypithecus francoisi | 107.4097278 | 22.59146389 |
| Trachypithecus francoisi | 107.4115028 | 22.58696667 |
| Trachypithecus francoisi | 107.4111278 | 22.58860556 |
| Trachypithecus francoisi | 107.4098083 | 22.58727222 |
| Trachypithecus francoisi | 107.4061722 | 22.58485278 |
| Trachypithecus francoisi | 107.4262583 | 22.56386111 |
| Trachypithecus francoisi | 107.42615 | 22.58614167 |
| Trachypithecus francoisi | 107.4266139 | 22.58502778 |
| Trachypithecus francoisi | 107.8198611 | 22.68652778 |
| Trachypithecus francoisi | 107.8196556 | 22.68587778 |
| Trachypithecus francoisi | 107.821875 | 22.69168333 |
| Trachypithecus francoisi | 107.7984583 | 22.67679167 |
| Trachypithecus francoisi | 107.7859167 | 22.74497778 |
| Trachypithecus francoisi | 107.7750139 | 22.74500556 |
| Trachypithecus francoisi | 107.7672083 | 22.74665 |
| Trachypithecus francoisi | 107.7611694 | 22.74982778 |
| Trachypithecus francoisi | 107.7578278 | 22.75342222 |
| Trachypithecus francoisi | 107.7528028 | 22.7487 |
| Trachypithecus francoisi | 107.751175 | 22.75277778 |
| Trachypithecus francoisi | 107.7536556 | 22.75365556 |
| Trachypithecus francoisi | 107.8316556 | 22.747475 |
| Trachypithecus francoisi | 107.8282639 | 22.74905278 |
| Trachypithecus francoisi | 107.8250139 | 22.75015 |
| Trachypithecus francoisi | 106.9467167 | 22.46438333 |
| Trachypithecus francoisi | 106.9451111 | 22.46304167 |
| Trachypithecus francoisi | 106.9442417 | 22.46338889 |
| Trachypithecus francoisi | 106.9394889 | 22.46453611 |
| Trachypithecus francoisi | 106.9427861 | 22.46352222 |
| Trachypithecus francoisi | 106.9365167 | 22.464575 |
| Trachypithecus francoisi | 106.9304861 | 22.46520833 |
| Trachypithecus francoisi | 106.9256417 | 22.46489167 |
| Trachypithecus francoisi | 106.9198417 | 22.46696944 |
| Trachypithecus francoisi | 106.9209694 | 22.46514722 |
| Trachypithecus francoisi | 106.904475 | 22.47513889 |
| Trachypithecus francoisi | 106.9055139 | 22.47336389 |
| Trachypithecus francoisi | 106.9106528 | 22.47407222 |
| Trachypithecus francoisi | 106.9135972 | 22.47290833 |
| Trachypithecus francoisi | 106.9194361 | 22.50094722 |
| Trachypithecus francoisi | 106.9123 | 22.501175 |
| Trachypithecus francoisi | 106.9102917 | 22.5009 |
| Trachypithecus francoisi | 106.8957139 | 22.49997222 |
| Trachypithecus francoisi | 106.9094056 | 22.48789444 |
| Trachypithecus francoisi | 106.9216167 | 22.49241944 |
| Trachypithecus francoisi | 106.9146389 | 22.49008056 |
| Trachypithecus francoisi | 106.9050611 | 22.48615833 |
| Trachypithecus francoisi | 106.9293167 | 22.46098333 |
| Trachypithecus francoisi | 106.9347583 | 22.46586667 |
| Trachypithecus francoisi | 106.937375 | 22.47098889 |
| Trachypithecus francoisi | 106.8267056 | 22.53885833 |
| Trachypithecus francoisi | 106.8435917 | 22.52517778 |
| Trachypithecus francoisi | 106.8554083 | 22.51671111 |
| Trachypithecus francoisi | 106.8554139 | 22.51670556 |
| Trachypithecus francoisi | 106.8611306 | 22.51538333 |
| Trachypithecus francoisi | 107.0023417 | 22.44406944 |
| Trachypithecus francoisi | 107.003925 | 22.44053333 |
| Trachypithecus francoisi | 107.0337056 | 22.41703333 |
| Trachypithecus francoisi | 104.6656389 | 25.57758333 |
| Trachypithecus francoisi | 104.6656111 | 25.57758333 |
| Trachypithecus francoisi | 104.6656667 | 25.57761111 |
| Trachypithecus francoisi | 104.6657222 | 25.57763889 |
| Trachypithecus francoisi | 104.63675 | 26.09805556 |
| Trachypithecus francoisi | 104.6361389 | 26.09916667 |
| Trachypithecus francoisi | 104.6363889 | 26.09861111 |
| Trachypithecus francoisi | 104.6366667 | 26.09827778 |
| Trachypithecus francoisi | 104.63675 | 26.09805556 |
| Trachypithecus francoisi | 107.0665278 | 28.17233333 |
| Trachypithecus francoisi | 107.0675278 | 28.16791667 |
| Trachypithecus francoisi | 107.0679722 | 28.167 |
| Trachypithecus francoisi | 107.0686111 | 28.16527778 |
| Trachypithecus francoisi | 107.0700278 | 28.16352778 |
| Trachypithecus francoisi | 107.0703333 | 28.16383333 |
| Trachypithecus francoisi | 107.0741111 | 28.18530556 |
| Trachypithecus francoisi | 107.0754444 | 28.18433333 |
| Trachypithecus francoisi | 107.0775278 | 28.18405556 |
| Trachypithecus francoisi | 107.0801389 | 28.18369444 |
| Trachypithecus francoisi | 107.0815 | 28.1835 |
| Trachypithecus francoisi | 107.082 | 28.18333333 |
| Trachypithecus francoisi | 107.0840833 | 28.18272222 |
| Trachypithecus francoisi | 107.0718333 | 28.21727778 |
| Trachypithecus francoisi | 107.0764722 | 28.21547222 |
| Trachypithecus francoisi | 107.0786389 | 28.21136111 |
| Trachypithecus francoisi | 107.0814167 | 28.21025 |
| Trachypithecus francoisi | 107.09125 | 28.20616667 |
| Trachypithecus francoisi | 107.0971389 | 28.20419444 |
| Trachypithecus francoisi | 107.1023611 | 28.20358333 |
| Trachypithecus francoisi | 107.4215833 | 29.06797222 |
| Trachypithecus francoisi | 107.4213056 | 29.06611111 |
| Trachypithecus francoisi | 107.4225278 | 29.06391667 |
| Trachypithecus francoisi | 107.4228611 | 29.06333333 |
| Trachypithecus francoisi | 107.4202778 | 29.07491667 |
| Trachypithecus francoisi | 107.4206389 | 29.07644444 |
| Trachypithecus francoisi | 107.4203056 | 29.07875 |
| Trachypithecus francoisi | 107.4206111 | 29.07902778 |
| Trachypithecus francoisi | 108.2314972 | 28.65844444 |
| Trachypithecus francoisi | 108.2104444 | 28.66001389 |
| Trachypithecus francoisi | 108.1830556 | 28.64638889 |
| Trachypithecus francoisi | 108.3222222 | 28.65045833 |
| Trachypithecus francoisi | 108.1351667 | 28.64208889 |
| Trachypithecus francoisi | 108.1193889 | 28.63183333 |
| Trachypithecus francoisi | 108.1001472 | 28.62747222 |
| Trachypithecus francoisi | 108.131475 | 28.66739444 |
| Trachypithecus francoisi | 108.1222833 | 28.68358611 |
| Trachypithecus francoisi | 108.1440056 | 28.69013611 |
| Trachypithecus francoisi | 108.1497583 | 28.68147778 |
| Trachypithecus francoisi | 108.1495 | 28.67455 |
| Trachypithecus francoisi | 108.161 | 28.66340278 |
| Trachypithecus francoisi | 108.1713861 | 28.67191389 |
| Trachypithecus francoisi | 108.2807361 | 28.88120556 |
| Trachypithecus francoisi | 108.2747278 | 28.86850278 |
| Trachypithecus francoisi | 108.2736111 | 28.86091111 |
| Trachypithecus francoisi | 108.2630528 | 28.84106389 |
| Trachypithecus francoisi | 108.2177361 | 28.79413889 |
| Trachypithecus francoisi | 108.2125861 | 28.76871111 |
| Trachypithecus francoisi | 108.1840889 | 28.777725 |
| Trachypithecus francoisi | 108.1619444 | 28.78549444 |
| Trachypithecus francoisi | 108.1834028 | 28.74749444 |
| Trachypithecus francoisi | 108.1883806 | 28.72188889 |
| Trachypithecus francoisi | 108.1804861 | 29.12776944 |
| Trachypithecus francoisi | 108.1587694 | 28.70413889 |
| Trachypithecus francoisi | 108.1902694 | 28.78180278 |
| Trachypithecus francoisi | 108.1511306 | 28.74056944 |
| Trachypithecus francoisi | 108.0707944 | 28.65624722 |
| Trachypithecus francoisi | 108.0683889 | 28.69028611 |
| Trachypithecus francoisi | 108.0227278 | 28.63967778 |
| Trachypithecus francoisi | 107.3762472 | 29.05719444 |
| Trachypithecus francoisi | 107.4292333 | 29.05678611 |
| Trachypithecus francoisi | 107.4140056 | 29.063275 |
| Trachypithecus francoisi | 107.41115 | 29.09513333 |
| Trachypithecus francoisi | 107.4298528 | 29.06074167 |
| Trachypithecus francoisi | 107.4371889 | 29.05325833 |
| Trachypithecus francoisi | 107.3998639 | 29.05824444 |
| Trachypithecus francoisi | 107.4002361 | 29.04508056 |
| Trachypithecus francoisi | 107.4039639 | 29.07644167 |
| Trachypithecus francoisi | 107.3998611 | 29.065 |
| Trachypithecus francoisi | 107.4027361 | 29.09243333 |
| Trachypithecus francoisi | 107.3752417 | 29.1582 |
| Trachypithecus francoisi | 107.3805444 | 29.174775 |
| Trachypithecus francoisi | 107.3890944 | 29.18698056 |
| Trachypithecus francoisi | 107.3598778 | 29.14722222 |
| Trachypithecus francoisi | 107.3852944 | 29.13785833 |
| Trachypithecus francoisi | 107.3804861 | 29.13642778 |

1. 19 bioclimatic variable data was downloaded from the WorldClim (www.worldclim.com) dataset. Digital Elevation Model (DEM) data was downloaded from geospatial data cloud (http://www.gscloud.cn). NDVI was downloaded from the Resources Environment Data Center, Chinese Academy of Sciences (http://www.resdc.cn). River, road and housing data were obtained from the National Basic Geographic Information System.
