# Supplementary material for: Predicting the potential distribution change of the endangered Francois' langur (Trachypithecus francoisi) across its entire range in China under climate change
Source: Ecol Evol. 2024 Jul 10;14(7):e11684. doi: 10.1002/ece3.11684 (PMC11236436; doi:10.1002/ece3.11684)

APPENDIXES

APPENDIX 1: Pearson’ s correlation coefficients between 19 climate variables, and intensity of color represents greater positive (blue) or negative correlation (orange).


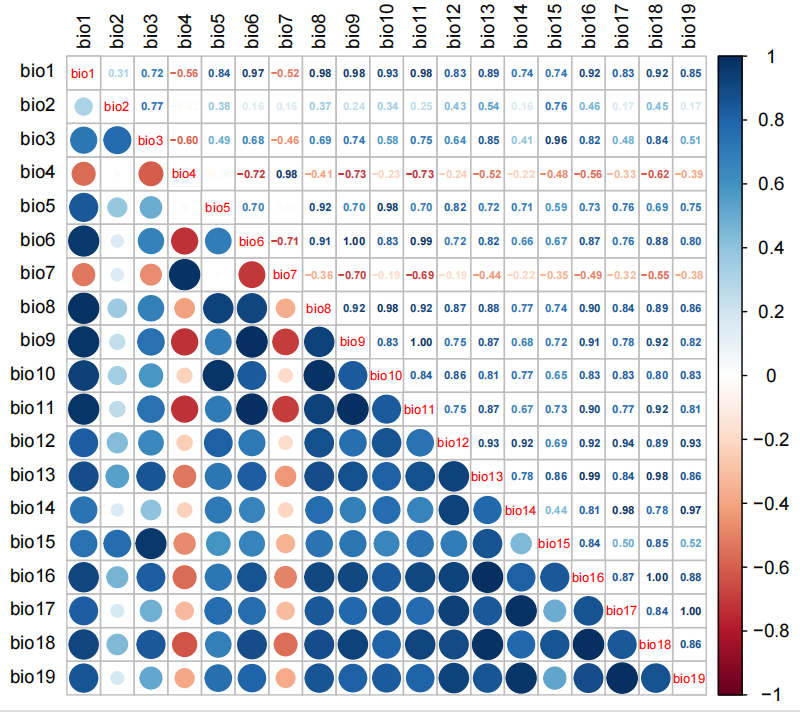


APPENDIX 2: The receiver operating characteristic (ROC) curve and average area under curve (AUC) values for the optimized model over 10 replicate runs were shown in red, while blue margins show ± standard deviation (SD) calculated for 10 replicates


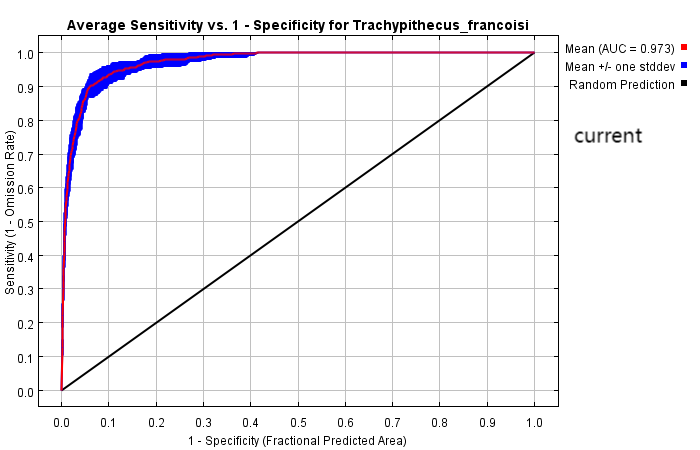


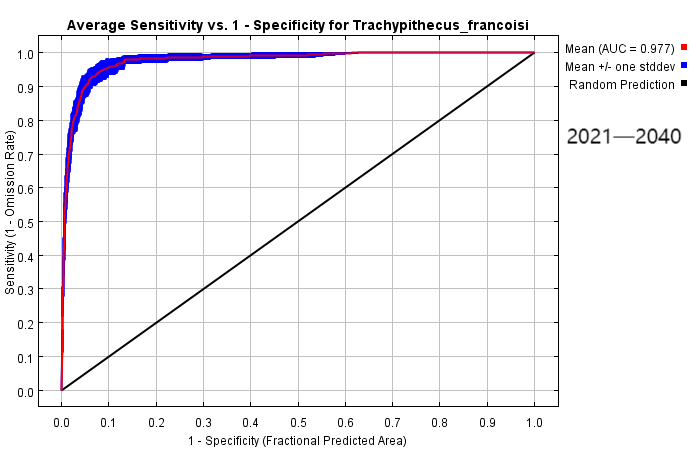


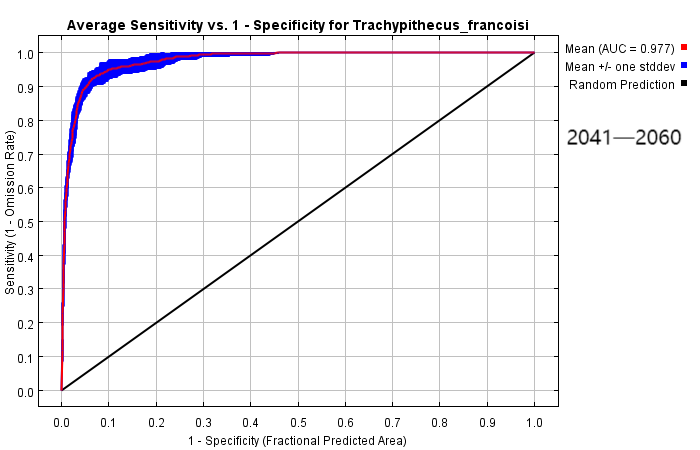


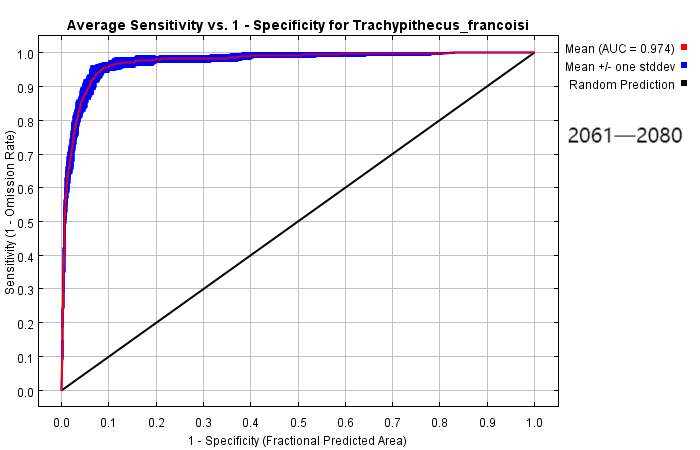


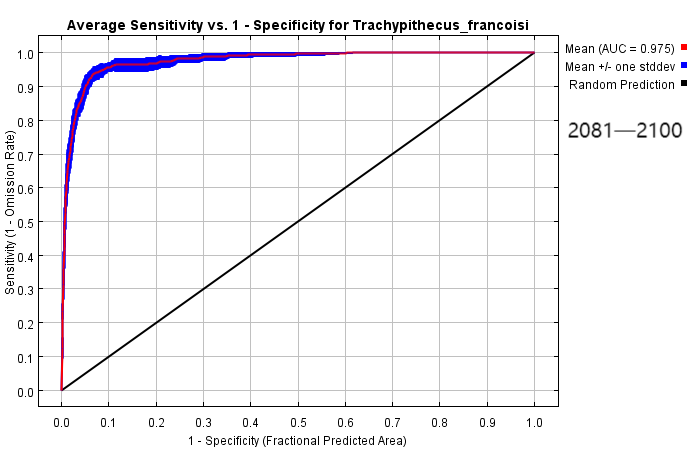


APPENDIX 3: Response curves of environmental variables in the potential distribution model of Francois' langur. Note: The red curves represent average value over 10 replicate runs, while blue margins represented ± SD calculated for 10 replicates


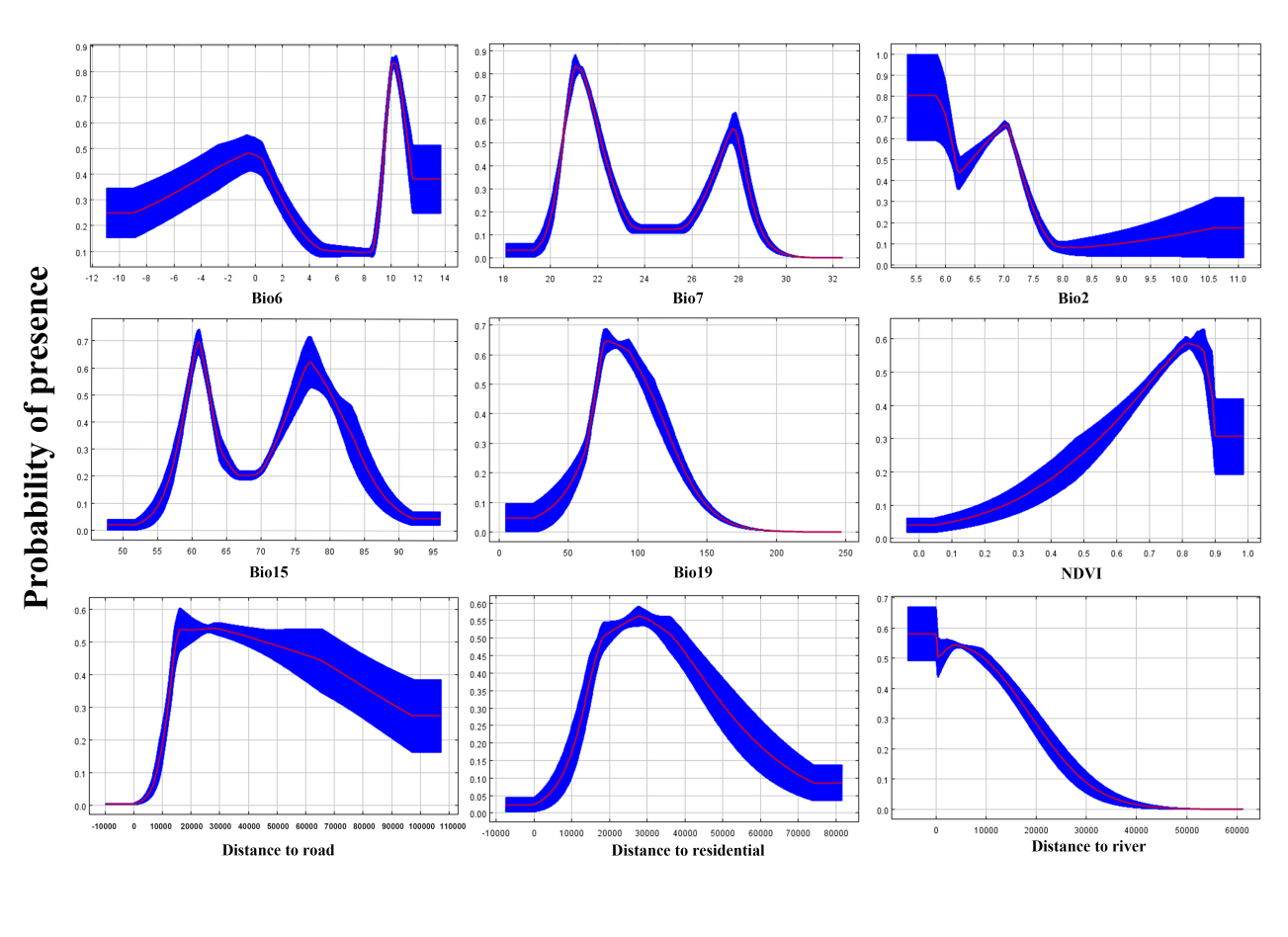


APPENDIX 4: Centroid change in the potential distribution of Francois' langur from 2021 to 2100. The picture on the left shows the northern population and the picture on the right shows the southern population.


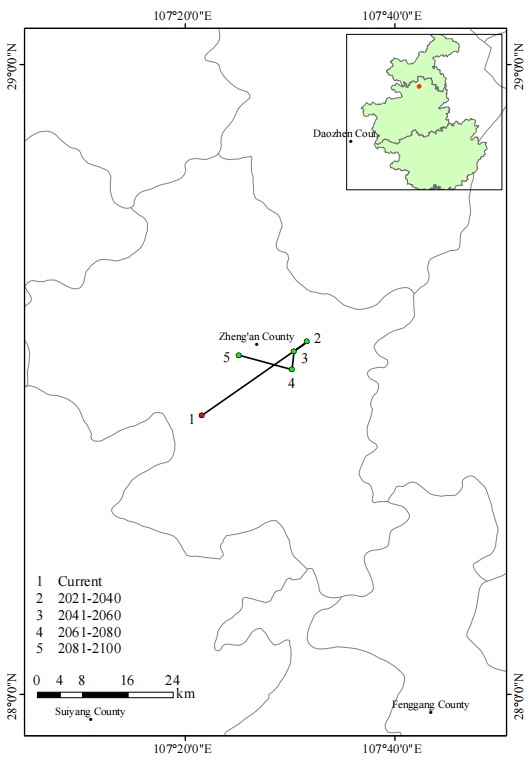

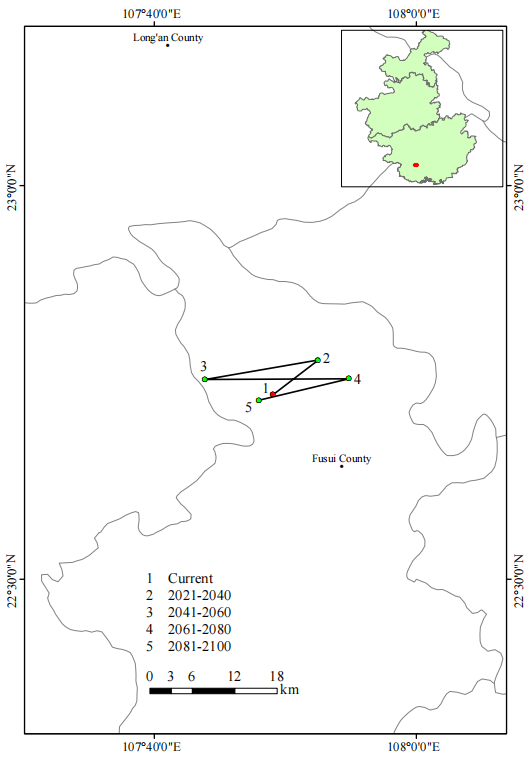

Supplement: Supplementary file 1 — Appendix S1. [file ECE3-14-e11684-s001.zip › Appendix caption.docx]
