# Supplementary figures and images for: Predicting the potential distribution change of the endangered Francois' langur (Trachypithecus francoisi) across its entire range in China under climate change
Source: Ecol Evol. 2024 Jul 10;14(7):e11684. doi: 10.1002/ece3.11684 (PMC11236436; doi:10.1002/ece3.11684)

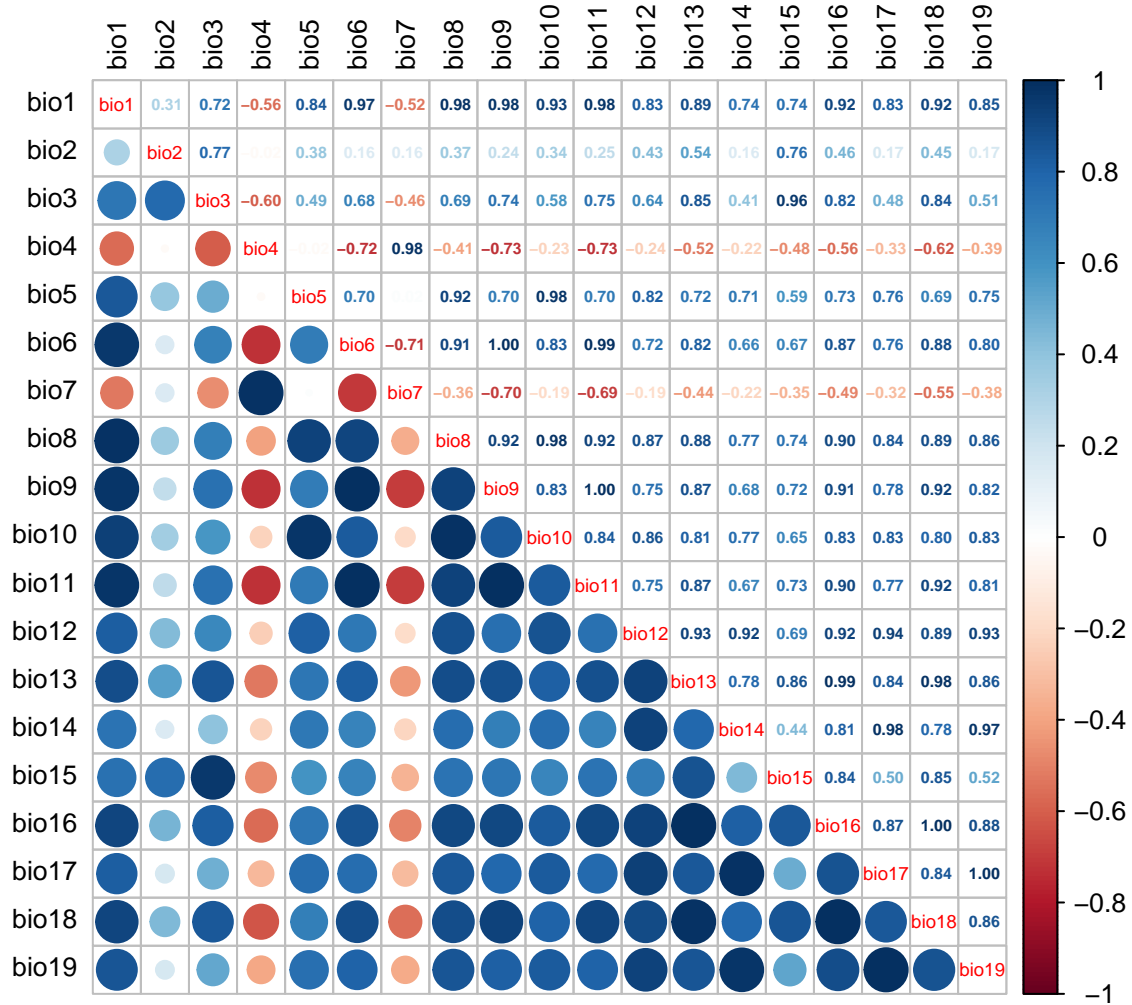

Supplement: Supplementary file 1 — Appendix S1. [file ECE3-14-e11684-s001.zip › APPENDIX 1.pdf]

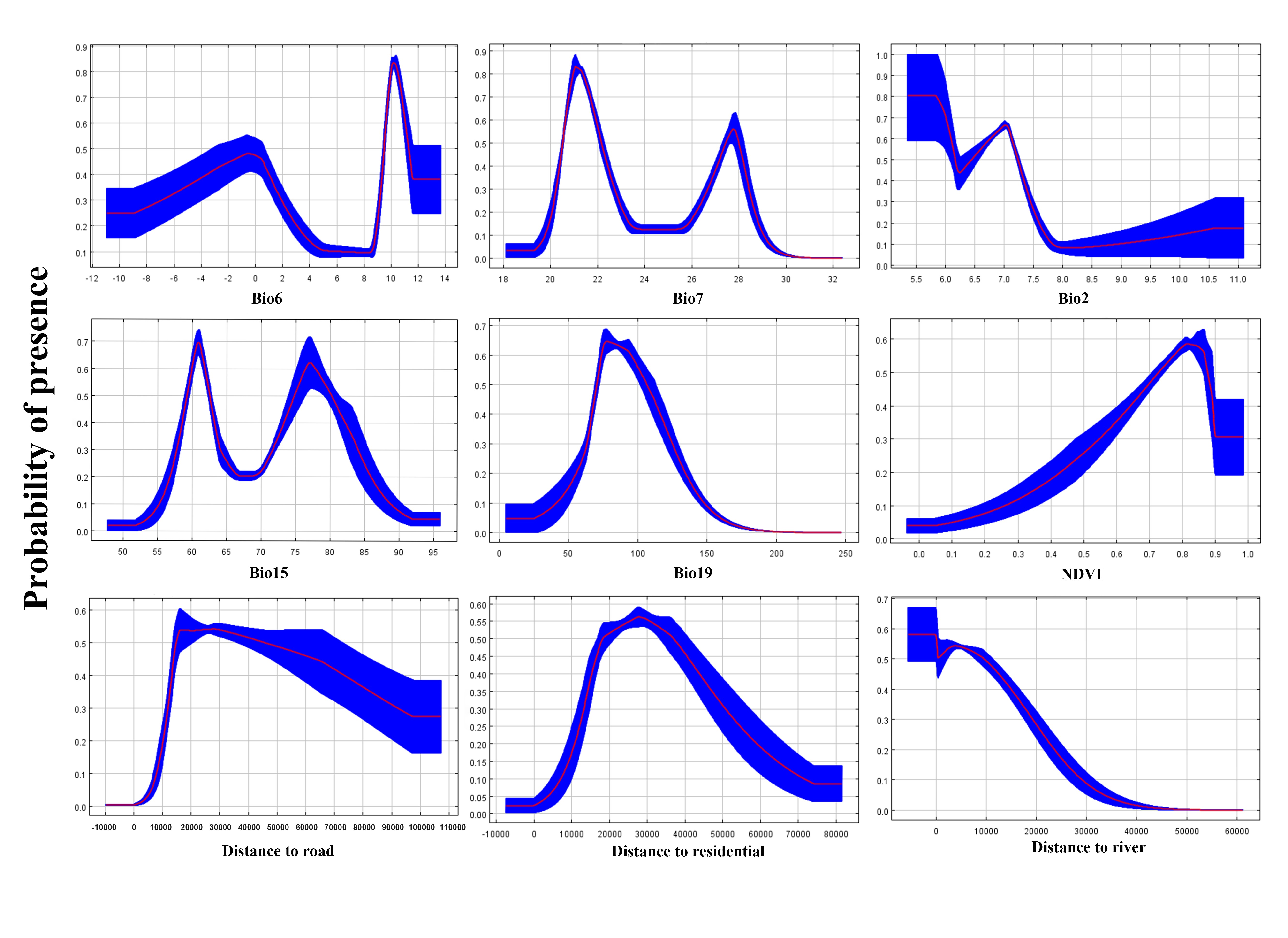

Supplement: Supplementary file 1 — Appendix S1. [file ECE3-14-e11684-s001.zip › APPENDIX 3.jpg]

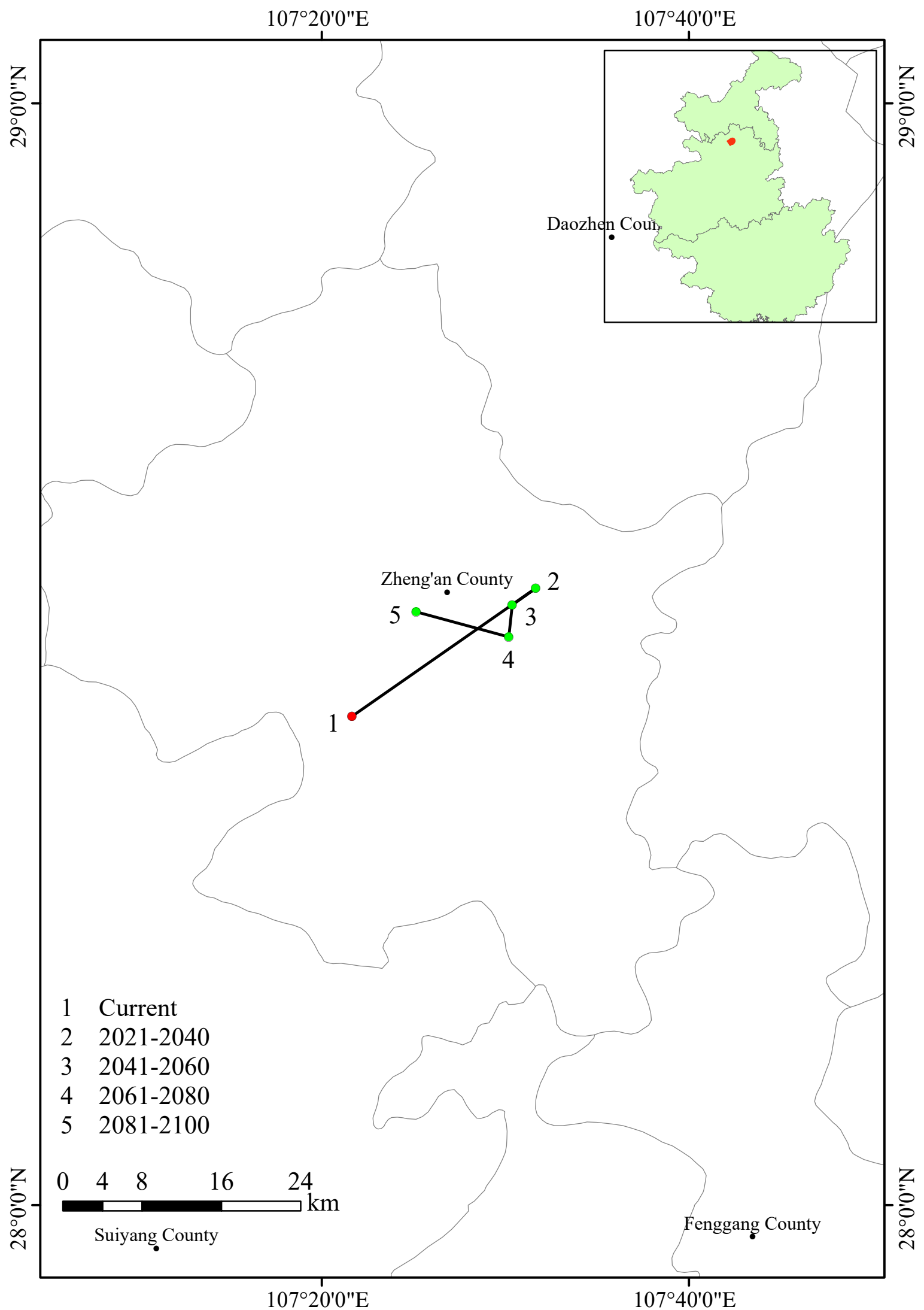

Supplement: Supplementary file 1 — Appendix S1. [file ECE3-14-e11684-s001.zip › APPENDIX 4-1.pdf]

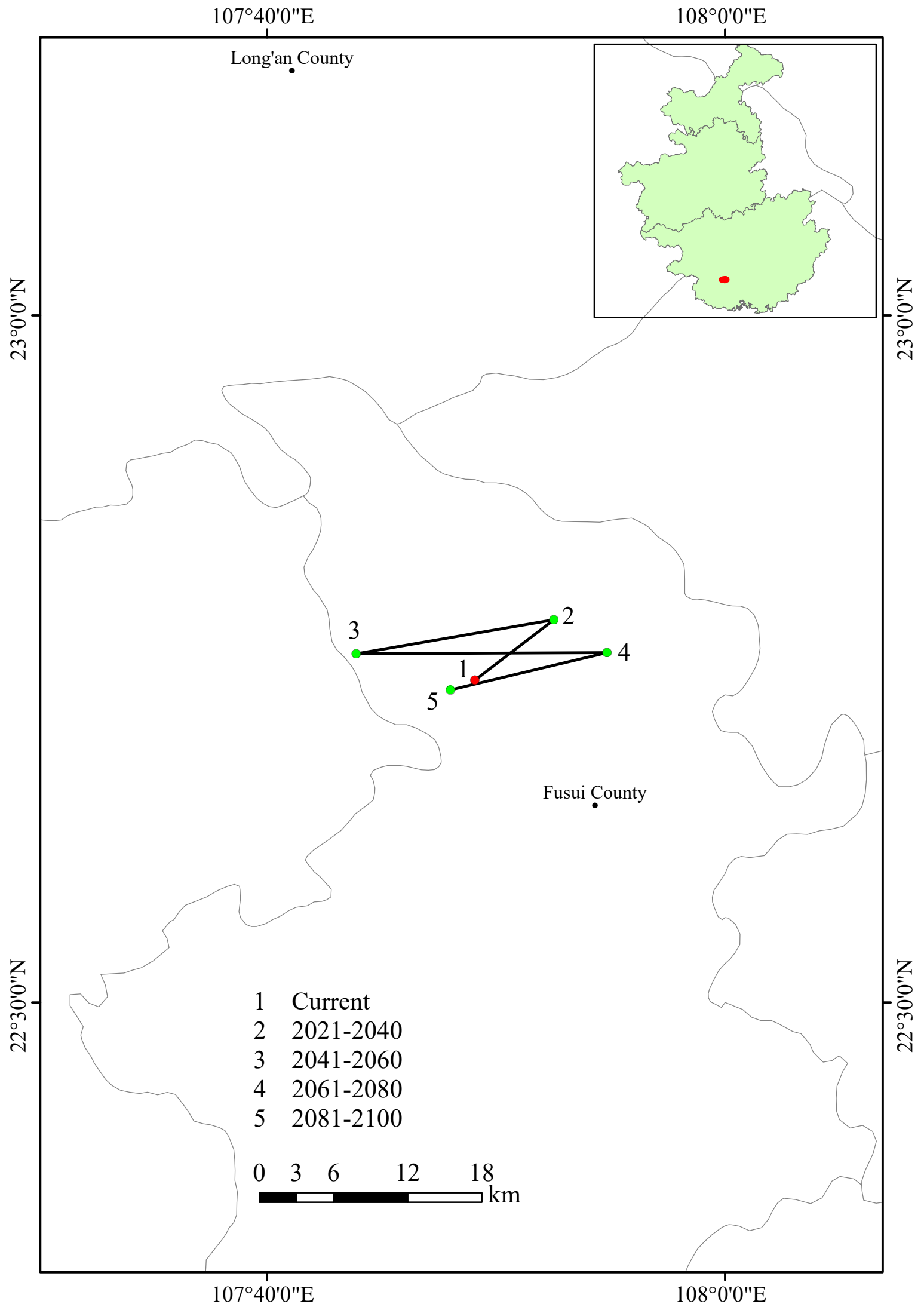

Supplement: Supplementary file 1 — Appendix S1. [file ECE3-14-e11684-s001.zip › APPENDIX 4-2.pdf]

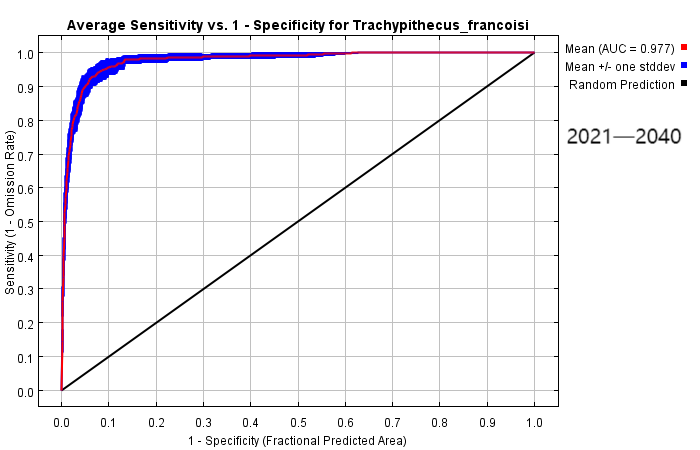

Supplement: Supplementary file 1 — Appendix S1. [file ECE3-14-e11684-s001.zip › Trachypithecus_francoisi_roc2040.png]

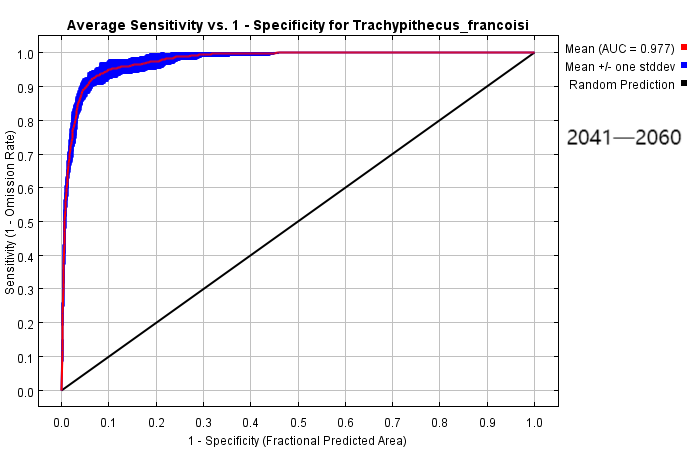

Supplement: Supplementary file 1 — Appendix S1. [file ECE3-14-e11684-s001.zip › Trachypithecus_francoisi_roc2060.png]

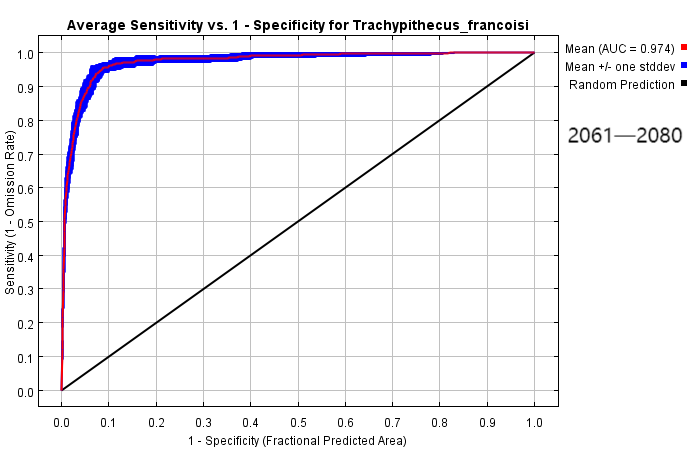

Supplement: Supplementary file 1 — Appendix S1. [file ECE3-14-e11684-s001.zip › Trachypithecus_francoisi_roc2080.png]

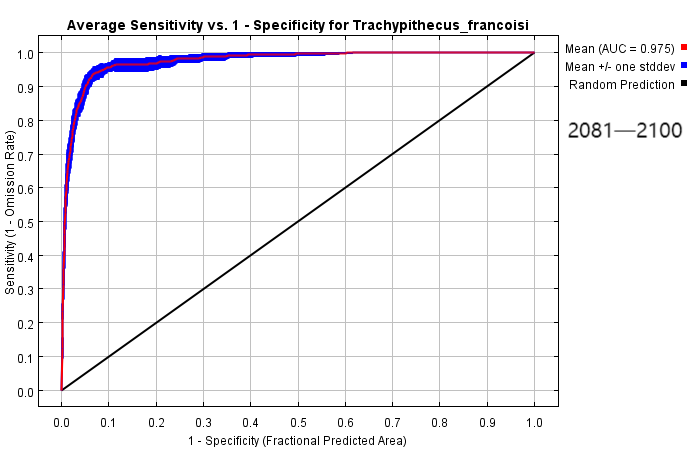

Supplement: Supplementary file 1 — Appendix S1. [file ECE3-14-e11684-s001.zip › Trachypithecus_francoisi_roc2100.png]

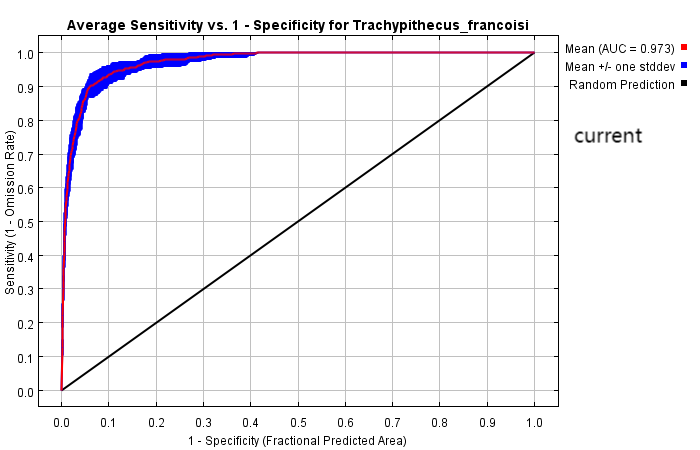

Supplement: Supplementary file 1 — Appendix S1. [file ECE3-14-e11684-s001.zip › Trachypithecus_francoisi_roc-current.png]
